# Left posterior temporal cortex is sensitive to syntax within conceptually matched Arabic expressions

**DOI:** 10.1038/s41598-021-86474-x

**Published:** 2021-03-30

**Authors:** Suhail Matar, Julien Dirani, Alec Marantz, Liina Pylkkänen

**Affiliations:** 1 Department of Psychology, New York University, New York, NY USA; 2 Department of Linguistics, New York University, New York, NY USA; 3 NYUAD Research Institute, New York University Abu Dhabi, Abu Dhabi, UAE

**Keywords:** Language, Reading

## Abstract

During language comprehension, the brain processes not only word meanings, but also the grammatical structure—the “syntax”—that strings words into phrases and sentences. Yet the neural basis of syntax remains contentious, partly due to the elusiveness of experimental designs that vary structure independently of meaning-related variables. Here, we exploit Arabic’s grammatical properties, which enable such a design. We collected magnetoencephalography (MEG) data while participants read the same noun-adjective expressions with zero, one, or two contiguously-written definite articles (e.g., ‘*chair purple*’; ‘*the-chair purple*’; ‘*the-chair the-purple*’), representing equivalent concepts, but with different levels of syntactic complexity (respectively, indefinite phrases: ‘*a purple chair*’; sentences: ‘*The chair is purple.*’; definite phrases: ‘*the purple chair*’). We expected regions processing syntax to respond differently to simple versus complex structures. Single-word controls (‘*chair*’/‘*purple*’) addressed definiteness-based accounts. In noun-adjective expressions, syntactic complexity only modulated activity in the left posterior temporal lobe (LPTL), ~ 300 ms after each word’s onset: indefinite phrases induced more MEG-measured positive activity. The effects disappeared in single-word tokens, ruling out non-syntactic interpretations. In contrast, left anterior temporal lobe (LATL) activation was driven by meaning. Overall, the results support models implicating the LPTL in structure building and the LATL in early stages of conceptual combination.

## Introduction

The neural basis of reading comprehension is multi-faceted and comprises many different computations, including the composition of individual words to build more complex phrases and sentences. This combinatory procedure involves processing syntax—i.e., the underlying hierarchical structure of a phrase or sentence. Correctly representing syntactic structures can be crucial for comprehension, since word meanings (e.g., ‘*mouse*’, ‘*cat*’, ‘*chase*’) and world knowledge (Cats chase mice.) alone may lead to incorrect interpretations (given a sentence such as ‘*The mouse chased the cat.*’).

But although syntax has been extensively studied theoretically and experimentally, we have yet to form a clear, coherent picture of the neural basis of syntactic processing. In fact, the range of hypotheses remains wide, extending from the assertion that a specific subpart of a frontal cortical area processes syntax specifically^1^, to the claim that syntactic processing is inseparable from processing meaning within a cortical ‘language network’^2,3^.

One likely reason for the slow progress is the difficulty in varying syntactic structure independently of other confounding variables. The syntactic structure of a stimulus depends on the words used, and while we could manipulate the underlying structure by changing the words, doing so usually also changes the meaning of the stimulus and its visual (or auditory) properties, among other factors. One popular approach that attempts to dissociate syntax from meaning uses word lists^4,5^—jumbled-up word sequences that discourage syntactic structure building (‘*chased the the cat mouse*’)—and pseudo-word stimuli^6–9^—i.e., well-formed tokens that are devoid of meaning (‘*The fouse chaled the yat.*’). However, such unnatural stimuli may engage brain computations and processes that are extraneous to real language comprehension. In addition, most studies chiefly use functional MRI (fMRI) with its poor temporal resolution, obscuring the timing of syntactic effects.

**Figure 1:**
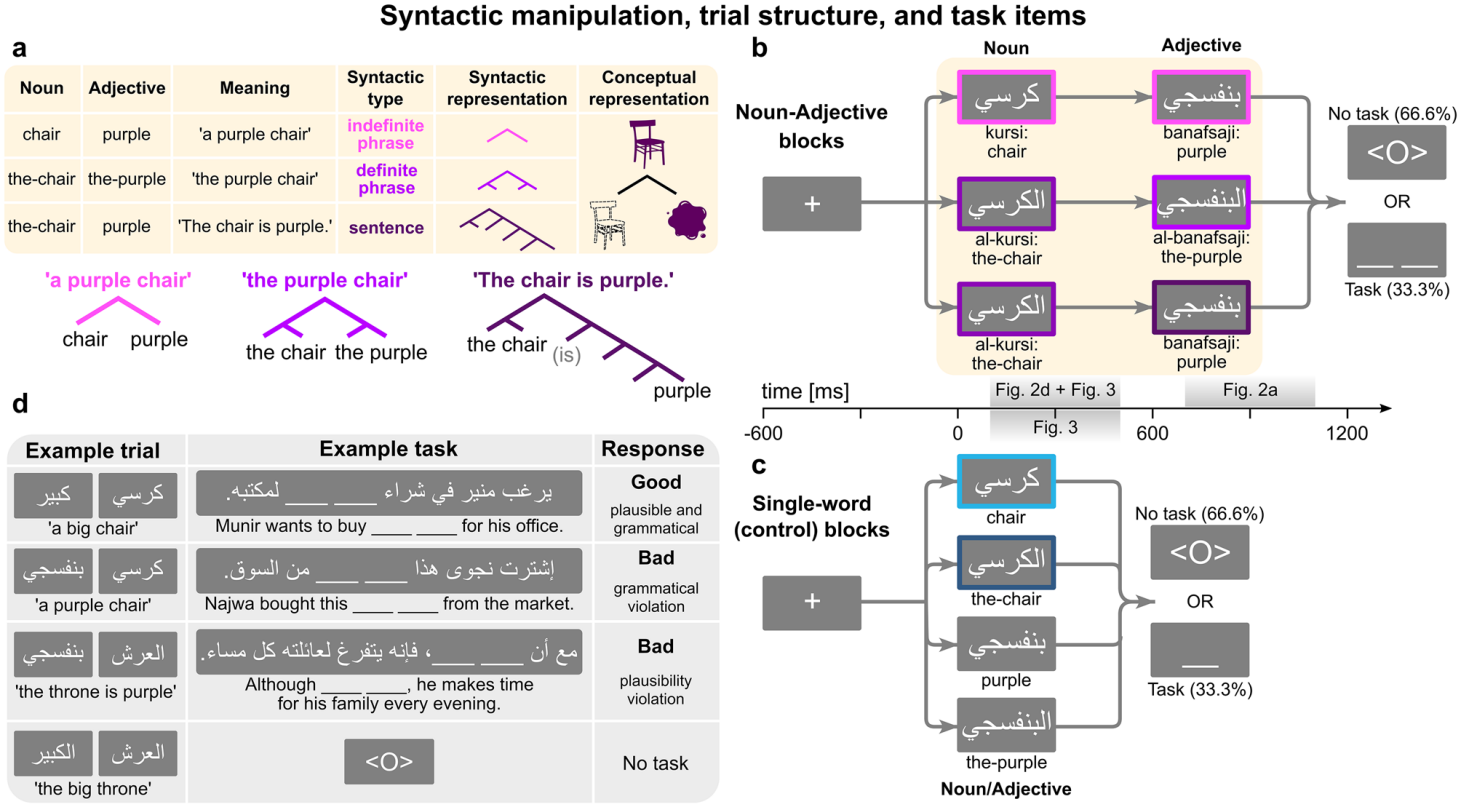
Syntactic manipulation and trial structure. (**a**) The three-way syntactic manipulation. Syntactic representation column shows schematic structure per condition; detailed schematic structures appear below the table. (**b**) Trial structure in noun-adjective blocks. Highlighted areas on time axis indicate time window of analyses, and the corresponding figure numbers. Colors correspond to those used in Figs. 2 and 3. (**c**) Trial structure of single-word control blocks. (**d**) Example task items.

Here, we use a two-pronged approach to elucidate the neural basis of syntax: (i) magnetoencephalography (MEG) recordings, with the dual benefits of millisecond temporal resolution and a good ability to spatially trace signals back to their cortical origins, and (ii) a Standard Arabic minimally-contrastive, two-word composition design, which manipulates syntactic structure independently of semantic, conceptual, and visual variables (Fig. 1a). To note, other studies have employed basic composition designs to investigate syntactic processing, but dissociated syntactic and semantic variables using either pseudo-words^1^ or determiner-noun (‘*this ship*’) versus adjective-noun (‘*blue ship*’) pairs^10^.

Just like English, Arabic adjectives are either attributive (‘a *purple* chair’) or predicational (‘The chair is *purple*.’). But unlike English, Arabic adjectives always follow their nouns, and they may bear the definite article (‘*al-*’), which is written inseparably from nouns/adjectives. As Fig. 1a shows, an adjective matching its noun’s definiteness (i.e., whether it bears the definite article) is attributive, while a bare adjective following a definite noun forms a predicate, creating a full sentence.

Though the three noun-adjective conditions in Fig. 1a feature the same words that build the same concept, the simple addition or removal of definite articles produces syntactic structures of different complexities. There are different ways to conceptualize syntactic structure. One way is to consider ‘offline’ structure—i.e., the syntactic structure of the whole expression (schematically shown in Fig. 1a)^11^; as this structure requires reading the whole expression, its complexity is only relevant after reading the second (and last) word. In our case, zero definite articles (‘*chair purple*’) produce the simplest syntactic structure—an indefinite phrase—while two articles (‘*the-chair the-purple*’) produce a definite phrase, with a more complex structure to accommodate the definite articles. (Moreover, some syntactic accounts analyze such adjectival structures as reduced relative clauses^12^; intuitively, this definite phrase has the same meaning as ‘*the chair that is purple*’.) The condition with only one article (‘*the-chair purple*’) corresponds to a full sentence and a more complex structure, which accommodates extra levels of syntactic information, such as tense (the chair is purple *now*). Thus, our design also decouples ‘offline’ syntactic complexity from mere visual form complexity. Note that we do not pre-suppose specific syntactic operands (e.g., Merge^1,13^, or Unification^14^); we simply assume indefinite phrases are less complex than the other conditions, because they feature less layers of information.

We can also consider what occurs during ‘online’ processing, as the brain builds the structure incrementally starting with the first word^11^. Recent evidence suggests that during comprehension, sensorially-driven bottom-up processes interact with top-down syntactic information^15–17^. In our case, indefinite nouns always lead to simple indefinite phrases, whereas definite nouns could lead to sentences or definite phrases, resulting either way in more complex structures. Therefore, definite nouns may be involved in predicting or projecting bigger structures than indefinite nouns on the first word.

**Figure 2:**
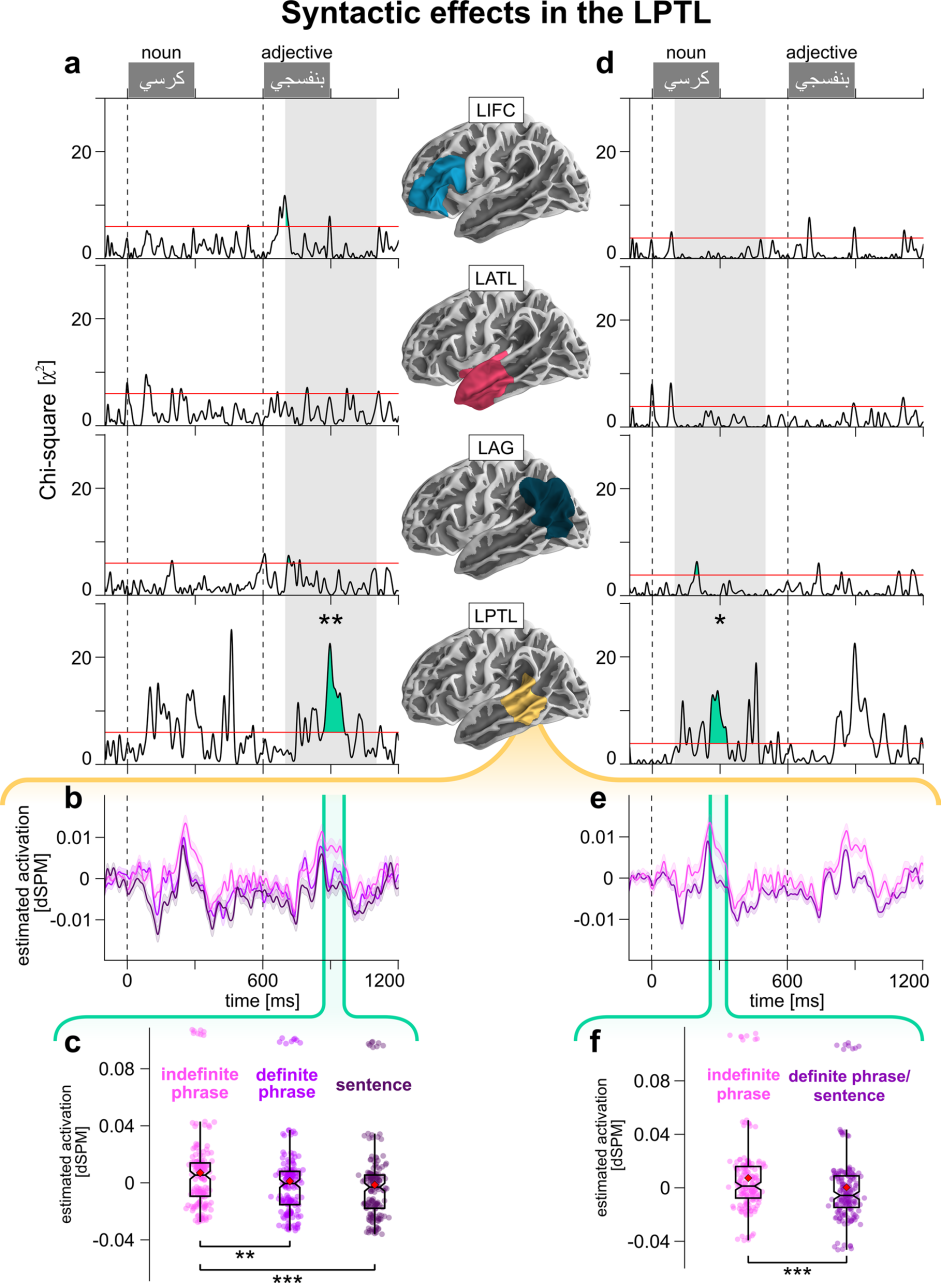
Results of syntactic effects analysis. (**a**) Results of likelihood ratio tests, comparing models with and without the syntactic factor for each cortical ROI. Gray bar indicates test time window (700–1100 ms). Horizontal red lines indicate cluster-forming thresholds. Filled green areas indicate suprathreshold clusters. Asterisks indicate significance of cluster-based permutation tests within ROI and test window. (**b**) Model-based estimated marginal means of LPTL activation per condition. Green area demarcates LPTL cluster in (**a**). Light bands indicate standard error of mean. (**c**) Estimated marginal means of LPTL activation per condition within the cluster in (**a**). Significance bars correspond to corrected pairwise comparisons. (**d**) Model comparison results assessing effect of noun definiteness in noun window (100–500 ms). Red lines indicate cluster-forming threshold. (**e**) Estimated marginal means of LPTL activation per condition. Green area shows LPTL cluster in (**d**). (**f**) Estimated marginal means within LPTL cluster in (**d**).

While participants read such noun-adjective pairs in a rapid serial presentation (Fig. 1b), we acquired MEG signals from sensors surrounding their heads. For all ROI analyses, we used single-trial regression and mixed effects models; we compared models with and without our factors of interest, to test whether our factors’ presence explains significantly more of the neural data. We focused on four left-hemispheric cortical regions most commonly associated with syntactic processing (Fig. 2a): the left anterior temporal lobe (LATL)^4,9,18,19^, the left posterior temporal lobe (LPTL; roughly corresponding to “Wernicke’s area”)^9,13,20–25^, the left inferior frontal cortex (LIFC; roughly corresponding to “Broca’s area”)^1,7,9,20,23,25,26^, and the left angular gyrus (LAG)^9,19^. We expected ROIs involved in syntactic processing to respond differently to the varying complexity of our conditions: if the ROI is involved in top-down predictions, we expected effects on the first word (the noun), and if it is involved in bottom-up syntactic combinatorics, we expected effects on the second word (the adjective). As to timing, recent MEG studies have shown syntactic effects a few hundred milliseconds after word onset^21,22^, so we chose a 100–500 ms test window after word onset.

Another set of electrophysiology studies has queried the effect of structure during comprehension on the coordinated firing of cohorts of neurons, which manifest as oscillations at different frequency bands. They found that *β*-band activity (roughly 10–30 Hz) increases more, compared to baseline, for structured (e.g., sentences) versus unstructured (e.g., word lists) stimuli^27,28^. To bridge ROI analyses with spectral oscillatory research, we compared the time–frequency representations of MEG sensor data in the *β*-band, expecting the more complex sentences and definite phrases to elicit greater activity increases in the *β*-band compared to simple indefinite phrases.

In separate blocks, participants read the same nouns and adjectives—with and without definite articles—but as single-word controls (Fig. 1c). These were important for ensuring the effects in two-word blocks were not merely due to the contrast between definite and indefinite words (see Results and Discussion). After one-third of the trials, a task sentence containing two blanks (or one, in single-word blocks) appeared: we instructed participants to mentally substitute the blank(s) with the word(s) of the most recent trial, and to indicate via button press whether or not the result is both grammatical and plausible (Fig. 1d; see Methods).

But reading comprehension is not limited to syntactic processing; the brain also engages in the crucial step of semantic composition—i.e., the process of building a complex concept (‘*purple chair*’) from individual ones (‘*purple*’, ‘*chair*’)^29^. A recent line of MEG research has implicated the LATL and its right-hemispheric homologue (RATL) in basic semantic composition. Several studies have shown that phrases (e.g., ‘*tomato dish*’) elicit more activation in these regions than single words (‘*dish*’), ~ 250 ms after noun onset^30–32^—including in Arabic^33^. Here, we used more properties of Arabic to further investigate semantic composition effects using two manipulations orthogonal to the syntactic factor (Fig. 5a).

We manipulated the *conceptual specificity* of the noun (e.g., low-specificity ‘*dish*’ vs. high-specificity ‘*soup*’), which affects behavioral^34^ and neural^35,36^ responses. In English, main nouns that have higher conceptual specificity (‘*tomato soup*’ vs. ‘*tomato dish*’) eliminate the LATL semantic composition effect; the same occurs with modifiers that have lower conceptual specificity (‘*vegetable dish*’ vs. ‘*tomato dish*’)^35,36^. However, it is unclear whether this depends on the word’s role (main noun vs. modifier) or its position (first vs. second word). Since word order in Arabic is reversed, if specificity effects depend on word role, we expected low- but not high-specificity nouns to elicit the LATL effect; if they depend on word order, we expected the opposite.

Finally, semantic composition effects occur relatively early, before brain responses associated with accessing lexical meaning^37^, suggesting the effects might be sensorially-driven. We hypothesized that orthographic *form typicality*^38^—i.e., the degree to which a word’s written form indicates its category (e.g., noun, adjective)—could provide cues facilitating early composition. Form typicality has already been shown to affect early occipitotemporal activity in MEG^39^ and EEG^40^ reading studies. To test whether it affects composition, we included an adjectival form typicality manipulation (see Methods). We expected high-typicality adjectives (i.e., adjectives that look adjectival) to facilitate early LATL effects, and low-typicality adjectives (adjectives that look ‘nouny’) to eliminate them.

## Results

### Behavioral results

Overall task accuracy for the 21 participants included in neural analyses (see Methods) was 90.02% (SD = 5.57%; all participants: 86.52%, SD = 8.54%). Participants averaged 92.38% (SD = 4.13%) on grammatical and plausible items, 83.49% (SD = 10.64%) on grammatical violation items, and 91.83% (SD = 8.82%) on plausibility violation items. Though results suggest grammatical violation items were more difficult, we had not normed task items for difficulty—we simply designed them to keep participants engaged. Additionally, during each trial, participants could not predict which item type (if any) will appear. Therefore, we did not analyze behavioral results further.

### Main syntactic ROI analyses

We first addressed whether our syntactic manipulation explains MEG-estimated activation in our four ROIs beyond what other variables explain. To that end, we analyzed two-word trials 700–1100 ms after trial onset (Fig. 2a; adjective window, Fig. 1b). At each timepoint, we modeled activation as follows (bold indicates predictors missing in reduced model; see Methods for details):

1 $${activation}_{ROI}(t)=intercept+{\varvec{c}}{\varvec{o}}{\varvec{m}}{\varvec{p}}{\varvec{l}}{\varvec{e}}{\varvec{x}}{\varvec{i}}{\varvec{t}}{\varvec{y}}+{specificity}_{noun}\times {typicality}_{adj}+{freq}_{noun}+{freq}_{adj}+\mathrm{log}\left({time}\right)+\left({intercept}|participant\right)$$

Since syntactic complexity has three levels (indefinite phrase, definite phrase, sentence), model comparison resulted in a timecourse of χ^2^(2)-distributed statistics (parameter indicates degrees of freedom). The biggest contiguous clusters of suprathreshold (> 95^th^-percentile) statistics appear in green (Fig. 2a). We found clusters in LPTL (867–964 ms from trial onset), LATL (791–802 ms), LIFC (700–715 ms), and LAG (708–723 ms). A cluster-based permutation analysis (Monte Carlo simulation) revealed a significant syntactic effect only in the LPTL (*p* = 0.0032, corrected; remaining ROIs: *p* = 0.603; see Methods). We plotted the model estimation of mean LPTL activation over time, per condition (Fig. 2b), and regressed average LPTL cluster activity using the full model; we estimated marginal means per condition and performed pairwise comparisons (Fig. 2c). This revealed significant differences between indefinite phrases and sentences (*p* = 1.7 · 10^–6^; corrected), and between indefinite and definite phrases (*p* = 0.0014; but sentences vs. definite phrases: *p* = 0.285), with more positivity for indefinite phrases compared to the other conditions.

We repeated the analysis in the 100–500 ms window (Fig. 2d), in which participants had only encountered the noun. Thus, we replaced complexity with noun definiteness (definite, indefinite; Fig. 1b), grouping sentences and definite phrases together. Model comparison produced χ^2^(1)-distributed statistics, with LPTL (254–334 ms) and LAG (188–207 ms) clusters. A Monte Carlo simulation revealed a significant effect only in the LPTL (*p* = 0.022; LAG: *p* = 0.917). Pairwise comparison of estimated average LPTL cluster activity revealed significantly more positivity for indefinite compared to definite nouns (*p* = 4.8 · 10^–5^; Fig. 2e,f).

We repeated these analyses in right-hemispheric ROIs (Supplementary Fig. S1). The adjective window had clusters in the right posterior temporal lobe (RPTL: 720–738 ms), right anterior temporal lobe (RATL: 864–875 ms), and right angular gyrus (RAG: 861–885 ms). However, the Monte Carlo simulation found no significant effects (RPTL, RAG: *p* = 0.245; RATL: *p* = 0.5). In the noun window, we found clusters in RPTL (121–138 ms), RATL (192–215 ms), and right inferior frontal cortex (RIFC: 382–394 ms), but the Monte Carlo simulation revealed no significant effects (RPTL, RATL, RIFC: *p* = 0.547).

### Follow-up LPTL analyses

One potential confound is the possibility that these LPTL effects merely reflect sensitivity to noun definiteness, since the conditions that feature definite nouns elicit different LPTL activation than those that feature indefinite nouns. To test this, we conducted two follow-up LPTL analyses using single-word controls (Fig. 1c). First, we analyzed two-word trials and single-noun trials, between 100 and 500 ms. The full model was:

2 $${activation}_{LPTL}(t)=intercept+{\varvec{b}}{\varvec{l}}{\varvec{o}}{\varvec{c}}{\varvec{k}}\times {{\varvec{d}}{\varvec{e}}{\varvec{f}}{\varvec{i}}{\varvec{n}}{\varvec{i}}{\varvec{t}}{\varvec{e}}{\varvec{n}}{\varvec{e}}{\varvec{s}}{\varvec{s}}}_{{\varvec{n}}{\varvec{o}}{\varvec{u}}{\varvec{n}}}+{specificity}_{noun}+{freq}_{noun}+\mathrm{log}\left({time}\right)+\left({intercept}|participant\right)$$

**Figure 3:**
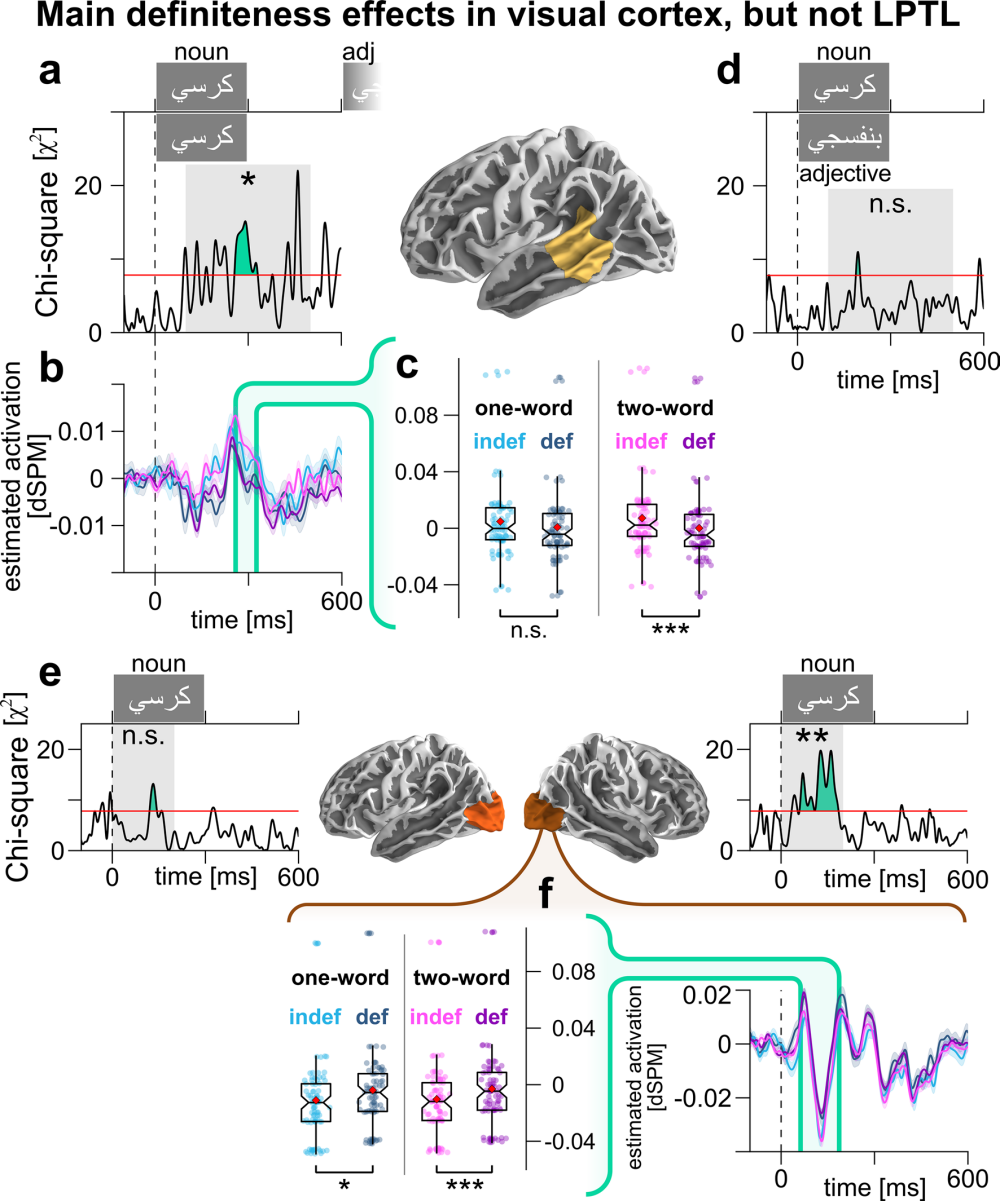
Follow-up analyses in the LPTL and visual cortex. (**a**) Model comparison results assessing the effect of noun definiteness, block type, and their interaction in the noun window. Red line indicates cluster-forming threshold. (**b**) Model-based estimated marginal means of LPTL per condition, with cluster from (**a**) shown in green. (**c**) Estimated marginal means of LPTL activation within cluster. Significance bars show corrected pairwise comparisons. (**d**) Model comparison results assessing the effect of word definiteness, syntactic category, and their interaction in single-word blocks. (**e**) Same as (**a**), but in the lateral occipital cortex, bilaterally, and in an earlier test window (0–200 ms). (**f**) Model-based estimated marginal means per condition over time (right) and averaged within cluster (left).

If LPTL effects were mere noun definiteness effects, we expected them regardless of block type (one-word, two-word). However, if the effects are syntactic, we expected a block type × definiteness interaction, with a definiteness difference in two-word blocks, exclusively. This is exactly what we found. Model comparison resulted in χ^2^(3)-distributed statistics, with an LPTL cluster (257–333 ms; Fig. 3a); the Monte Carlo simulation found a significant effect (*p* = 0.0214). We estimated marginal means per condition (Fig. 3b) and performed two pairwise comparisons: indefinite nouns had significantly more positivity than definite nouns in two-word (*p* = 4.7 · 10^–5^), but not single-word blocks (*p* = 0.151; Fig. 3c).

As a sanity check, and to ensure that we do find main definiteness effects (regardless of block type) where we expect them, we ran the same analysis between 0 and 200 ms in the lateral occipital cortex, bilaterally (Fig. 3e). We found a right lateral occipital cortex cluster (61–187 ms) and a significant effect there (*p* = 0.0056; left lateral occipital: 121–145 ms, *p* = 0.163). The underlying pattern shows more positivity for the definite nouns in both one-word (*p* = 0.0119) and two-word blocks (*p* = 2.7 · 10^–5^; Fig. 3f).

Secondly, we analyzed all single-word trials alone, to establish whether LPTL definite differences appear in either word category (nouns or adjectives), using this full model:

3 $${activation}_{LPTL}(t)=intercept+{{\varvec{c}}{\varvec{a}}{\varvec{t}}{\varvec{e}}{\varvec{g}}{\varvec{o}}{\varvec{r}}{\varvec{y}}}_{{\varvec{w}}{\varvec{o}}{\varvec{r}}{\varvec{d}}}\times {{\varvec{d}}{\varvec{e}}{\varvec{f}}{\varvec{i}}{\varvec{n}}{\varvec{i}}{\varvec{t}}{\varvec{e}}{\varvec{n}}{\varvec{e}}{\varvec{s}}{\varvec{s}}}_{{\varvec{w}}{\varvec{o}}{\varvec{r}}{\varvec{d}}}+{freq}_{word}+\mathrm{log}\left(time\right)+\left(intercept|participant\right)$$

We found an LPTL cluster (189–203 ms; Fig. 3d) of χ^2^(3)-distributed statistics, but the Monte Carlo simulation did not reveal significant effects there (*p* = 0.538).

### Spectral analysis

**Figure 4:**
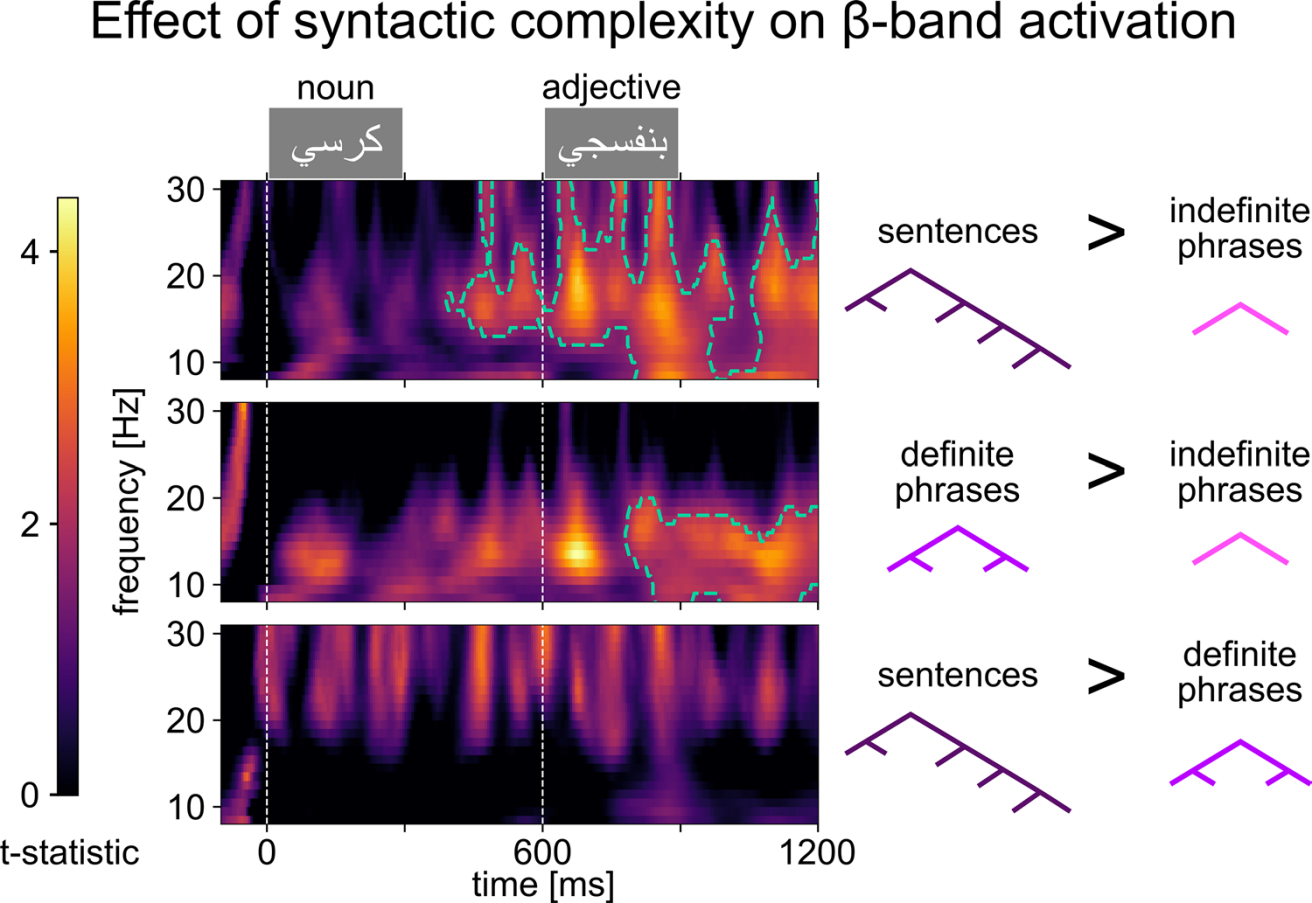
Pairwise comparisons of time–frequency representations of increased activity in two-word trials compared to baseline. Color bar shows *t* statistic values. White dashed lines represent word onsets. Green dashed lines delineate suprathreshold clusters which survive the permutation test.

We conducted three spectro-temporal clustering analyses on two-word trials in the time–frequency domain (see Methods). As hypothesized, we found clusters of increased *β*-band activation for the syntactically complex sentences (Fig. 4a) and definite phrases (Fig. 4b), both compared to the syntactically simple indefinite phrases (cluster extents: sentences > indefinite phrases, 390–1200 ms and 8–32 Hz; definite > indefinite phrases, 780–1200 ms and 8–20 Hz). The permutation test revealed significant differences in both comparisons (sentences vs. indefinite phrases: *p* = 0.0156; definite vs. indefinite phrases: *p* = 0.0302; FDR-corrected for three comparisons). The comparison between the two complex conditions (sentences > definite phrases) yielded clusters which did not survive the permutation test (Fig. 4c; smallest *p* = 0.366).

To ensure these effects are genuinely oscillatory (not simply evoked phase-locked components), we performed the same analysis on evoked response time–frequency representations (see Methods)^27^. No clusters in any of the three comparisons survived the permutation test (Supplementary Fig. S2; smallest clusters: sentences > indefinite phrases, *p* = 0.7; definite phrases > indefinite phrases, *p* = 0.837; sentences > definite phrases, *p* = 0.165). Qualitative comparison shows that the clusters delineated in the single-trial analyses do not appear in ERP comparisons.

### Semantic composition

**Figure 5:**
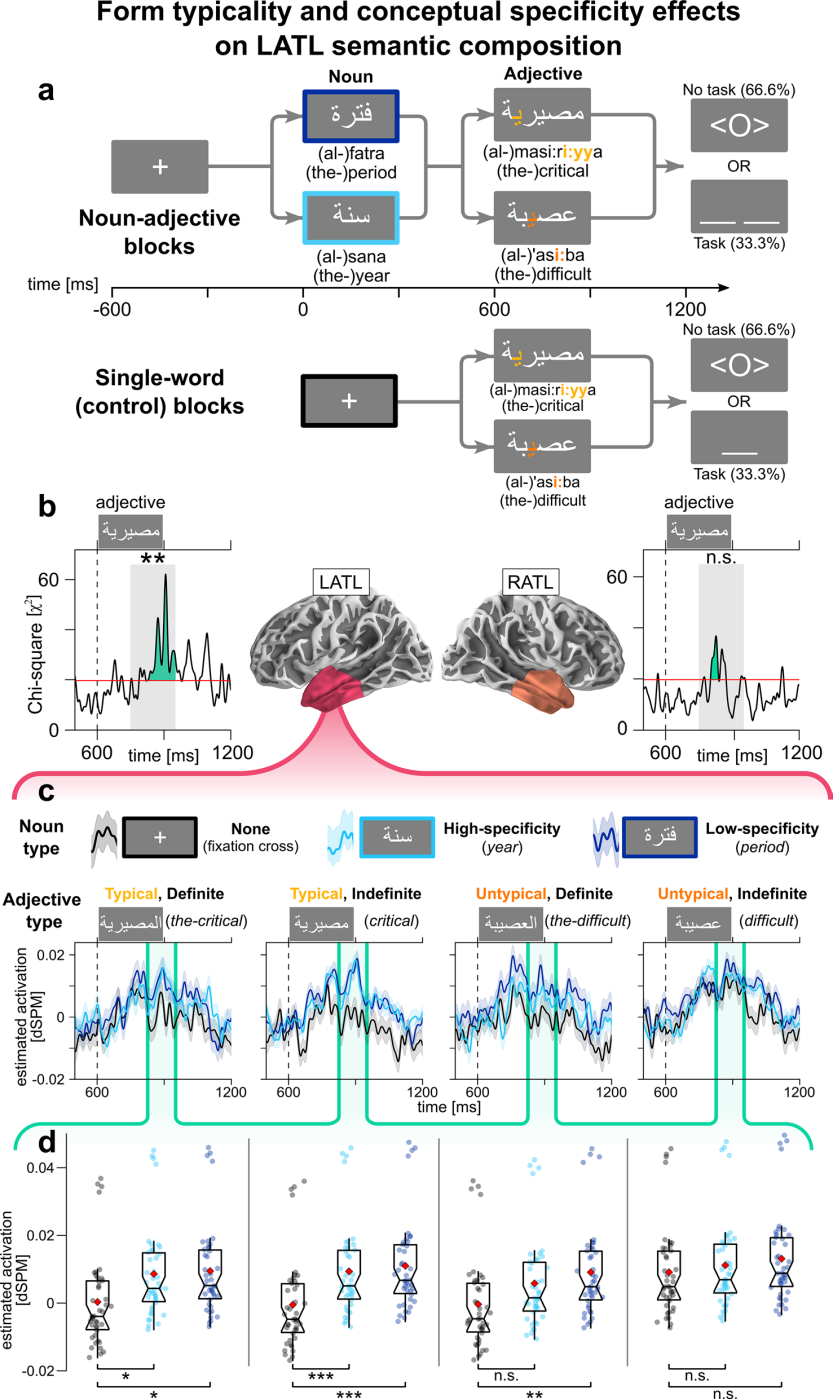
Semantic composition analyses and LATL effects of form typicality and conceptual specificity. (**a**) Trial structure outlining noun conceptual-specificity and adjectival form typicality manipulations. Highlighted letters on adjectives indicate form typicality (yellow) and untypicality (orange) cues. (**b**) Model comparison results assessing the effect of noun type, adjectival form typicality, adjectival definiteness, and all possible interactions in the adjective window (750–950 ms), in the LATL (left) and RATL (right). Red lines indicate cluster-forming threshold. (**c**) Model-based estimated marginal means of LATL activation per condition. Conditions are separated by adjective form typicality and definiteness. Green shape demarcates extent of LATL cluster in (**a**). (**d**) Estimated marginal means of LATL activation within cluster per condition. Significance bars show corrected pairwise comparisons.

For the analysis of semantic composition effects in the anterior temporal lobe and its interaction with noun specificity and form typicality, we analyzed two-word trials and single-word adjectives between 750 and 950 ms (adjective window; Fig. 5a), using this full model:

4 $${activation}_{ROI}(t)=intercept+{{\varvec{t}}{\varvec{y}}{\varvec{p}}{\varvec{e}}}_{{\varvec{n}}{\varvec{o}}{\varvec{u}}{\varvec{n}}}\times {{\varvec{t}}{\varvec{y}}{\varvec{p}}{\varvec{i}}{\varvec{c}}{\varvec{a}}{\varvec{l}}{\varvec{i}}{\varvec{t}}{\varvec{y}}}_{{\varvec{a}}{\varvec{d}}{\varvec{j}}}\times {{\varvec{d}}{\varvec{e}}{\varvec{f}}{\varvec{i}}{\varvec{n}}{\varvec{i}}{\varvec{t}}{\varvec{e}}{\varvec{n}}{\varvec{e}}{\varvec{s}}{\varvec{s}}}_{{\varvec{a}}{\varvec{d}}{\varvec{j}}}+{freq}_{adj}+\mathrm{log}\left(time\right)+\left(intercept|participant\right)$$

Noun type had three levels (high-specificity, low-specificity, or none—i.e., single-word adjectives) and adjective form typicality two levels (high, low). Model comparison produced timecourses of χ^2^(11)-distributed statistics, with LATL (826–950 ms) and RATL clusters (803–840 ms; Fig. 5b). The Monte Carlo simulation found an LATL effect (*p* = 0.0016; RATL: *p* = 0.1143).

We estimated mean LATL activation levels over time, per condition. For visualization purposes, we divided the 12 conditions by adjective typicality and definiteness (Fig. 5c). We regressed averaged LATL cluster activity against the full model to estimate marginal means per condition. For each level of adjective typicality and definiteness, we performed pairwise comparisons between the three noun types: we wanted to know which circumstances drive differences between one- and two-word trials. Results show a three-way interaction between noun type, adjective typicality, and adjective definiteness (Fig. 5d; Supplementary Table S2). High form-typicality adjectives in two-word trials elicited more LATL positivity than single-word trials, regardless of adjective definiteness or noun specificity. In low form-typicality adjectives, the effect appeared only for definite adjectives, when comparing single-word and low noun-specificity trials.

## Discussion

We used MEG data and a minimally-contrastive basic composition design to investigate the neural basis of syntax. Of the four chosen ROIs, only the LPTL was sensitive to our syntactic manipulation in noun-adjective pairs, ~ 300 ms after onset of each word, with more positivity for the structurally-simple indefinite phrases compared to sentences and definite phrases. Here, we discuss the effect’s pattern and possible interpretations, and its locus in space and time.

The results rule out several interpretations. First, visual differences between conditions cannot explain the effect: sentences and indefinite phrases feature identical indefinite adjectives (Fig. 1a) but produced the biggest pairwise difference on the adjective (Fig. 2c).

Secondly, if noun definiteness (‘*the* chair’ in sentences and definite phrases, versus ‘*a* chair’ in indefinite phrases) explained LPTL activity, we would have observed similar effects in single-word nouns. The mere presence of noun-definiteness sensitivity in the LPTL might not necessarily preclude a syntactic role for the LPTL, though it would introduce a potential confound in the interpretation of LPTL results. However, our analysis rules out this possibility, since the LPTL difference disappears in single-word trials, regardless of word category (Fig. 3d). Also, nouns in two-word blocks, but not single-word blocks, elicited LPTL effects (Fig. 3a–c). As a sanity check, the right visual cortex did respond to noun definiteness across block types (Fig. 3e–f).

A remaining possibility is that the LPTL processes noun definiteness differently for multi-word and single-word contexts. But LPTL clusters are time-locked to each word’s onset (Fig. 2a,d). If adjective-window activity reflected the *noun*’s definiteness, the adjective-window cluster could have appeared at any different latency. It is more likely that the adjective cluster is a bottom-up response to the adjective itself.

The series of analyses we conducted rule out non-syntactic interpretations of the LPTL effects; the remaining viable possibility is that the effects reflect a syntactic computation, though its nature remains unclear. One possibility we consider is that, rather than complexity, LPTL activity simply tracks some function of syntactic transition probabilities between words within trials (e.g., the probability of encountering a definite adjective given an indefinite noun, *P(definite adjective | definite noun)*). To investigate this, we briefly consider three popular information theoretic metrics, expressed as a function of transition probabilities: entropy, entropy reduction, and surprisal. To compute these metrics, we calculated transition probabilities based on both the Arabic GigaWord corpus (e.g., in the corpus as a whole, what is *P(definite adjective | definite noun)*?) and our experimental design (e.g., in our experiment, specifically, what is *P(definite adjective | definite noun)*?; Fig. 1b).

Syntactic entropy reflects uncertainty about future syntactic information based on current and past information^41^. But on the adjective, corpus-extracted entropy values are similar across our three conditions (Supplementary Table S3). If LPTL activity reflects design-based entropy instead, we should have observed no differences between the three conditions in response to the adjective, since entropy becomes zero (trial is over).

The same applies for syntactic entropy *reduction*^42^: corpus values are zero for both definite and indefinite nouns (Supplementary Table S4), despite observed differences in the noun-window LPTL activity. As to design-based entropy reduction, indefinite nouns in two-word blocks, which invariably produce indefinite phrases, entail a bigger entropy reduction than definite nouns (Fig. 1b); but if this reduction explained LPTL activity, we would have observed the opposite response to the adjective—more positivity for sentences and definite phrases, as entropy reduction increases in these two conditions.

Surprisal (unlikelihood of current information, given past information)^41^ also fails to explain our results. On the adjective, corpus-based surprisal is highest for sentences and lowest for definite phrases (Supplementary Table S5), which does not match the adjective LPTL pattern. As to design-based surprisal, in two-word blocks, indefinite nouns are less frequent (one in every three trials, Fig. 1b), thus more surprising. But if surprisal explained LPTL activity, we would have observed the opposite response to the adjective: given an indefinite noun, an indefinite adjective presents zero surprisal, whereas given a definite noun, definite and indefinite adjectives are equally more surprising.

Having ruled out syntactic interpretations that are not due to structure complexity, we turn to the remaining viable interpretations. On the one hand, we expected an ROI tapping into the ‘offline’ syntactic complexity of the whole expression to respond differently to complex versus simple stimuli during the presentation of the *second* word. On the other hand, we expected an ROI involved in predictive syntactic processing to respond differently to the same manipulation on the *first* word. Both predictions were borne out, both in the LPTL. Thus, it is possible that the two LPTL effects, after first word onset and after second word onset, reflect different syntactic processes. Specifically, it is unlikely that the LPTL effect after the first word (Fig. 2a–c) reflects purely bottom-up syntactic structure building, else, we would have observed the same activity patterns in response to nouns in one- and two-word trials (Fig. 3a). In two-word trials, it is rather the anticipation of a second word that likely elicits the noun effect. This is consistent with previous work showing that syntactic processing during comprehension is predictive^17,43–45^. Conversely, it is unlikely that the LPTL effect after the second word (Fig. 2d–f) is a top-down predictive process, since all trials ended after the second word, and the probability of a task item (and the type of the task item) was equal for all trials. The second word effect likely reflects bottom-up syntactic structure building or combinatorics.

Thus, the main remaining plausible possibility is that the LPTL engages in a dual syntactic role: a top-down predictive structure building on the noun (in two-word blocks, definite nouns trigger the prediction of a bigger, albeit underdetermined, syntactic structure compared to indefinite nouns)^11^, and a bottom-up syntactic composition process on the adjective (in two-word blocks, sentences and definite phrases trigger bottom-up building of bigger syntactic structures). This possible duality is reminiscent of and compatible with left-corner syntactic parsing strategies, which combine characteristics from both bottom-up and top-down parsers^46,47^. Moreover, the apparent involvement of LPTL in syntactic processing aligns with different models of syntactic processing in the brain, such as the prediction-binding model^48^, or the hierarchical structuring versus morpho-syntactic linearization model^23^, which ascribes the former to the LPTL.

Note that, throughout this work, we made no assumptions about the elementary operation at the heart of the syntactic computation (e.g., Merge^1,13^, Unification^14^, or otherwise). If LPTL activity indeed reflects syntactic structure processing, we do not make any specific claims about whether it tracks the number of elementary operations, or some other function describing the complexity of the syntactic representation; these different metrics are likely highly correlated in our design, and it was not our intention to adjudicate between them.

Regarding the effect’s locus, our results are in line with previous fMRI^9,24,25^, lesion^49^, and MEG^8,21,22^ findings implicating the LPTL in syntactic processing. However, they contrast with results from two main sets of studies in recent years. One set of results has implicated the LIFC in syntactic structure building (embodied by the theoretical operation Merge)^1,7,10,13^, while the other has presented evidence suggesting that syntactic processing is distributed across a large language network, and that it is inseparable from lexico-semantic processing^2,3^. These differences might be explainable in terms of experimental design. In a fully grammatical, lexico-conceptually and visually controlled design, we found that our syntactic manipulation did not significantly affect the LATL, LIFC, or LAG, suggesting a separation between the processing of syntax and meaning in the language network. Particularly interesting is the absence of LIFC effects, as this region has been traditionally associated with syntactic processing. However, recent findings suggest that production—rather than comprehension—demands drive activity in that region^23,24^. Our results also suggest left-lateralization of syntactic processing, as no right-hemispheric ROIs responded to our manipulation (Supplementary Fig. S1).

Our cluster timing (~ 300 ms post onset) comes after the 170-ms mark for *morphological decomposition*^50,51^—i.e., the step of breaking a complex written word (e.g., ‘*al-banafsaji:*’) down to its parts (‘*al*-’, ‘*banafsaji:*’). This suggests that by the time the clusters occur, information about definiteness and, by extension, the syntactic structure, is already available to the brain.

We also note that other MEG studies implicating the LPTL in syntactic processing reported earlier (~ 220 ms)^21^ and later (~ 350 ms)^22^ clusters; however, they used different designs with lengthier stimuli. Insofar as all LPTL effects tap into the same syntactic computations, this could suggest temporal flexibility in syntactic processing.

We also bridged ROI analyses with neural oscillations research. Our spectro-temporal analyses in two-word trials showed significantly larger increases in *β*-band activation for the syntactically more complex conditions (sentences, definite phrases) compared to the simple condition (indefinite phrases; Fig. 4a–b). This extends previous results showing such *β*-band effects when comparing stimuli with and without syntactic structure^27^. Here, we replicate this between equally grammatical stimuli that, while visually and conceptually matched, have different levels of syntactic complexity. Our results support models assigning lower *β*-band oscillations a major role in syntactic processing^48^. Importantly, both clusters (Fig. 4a–b) cover the temporal extent of the LPTL clusters after the second word in the ROI analyses (~ 900 ms). The clusters largely appear after the onset of the second word, even though ROI analyses revealed effects after the first word, too. This could further support the speculation that the two LPTL effects underlyingly reflect different processes, only one of which (bottom-up structure building) appears in *β*-band activity. Cross-frequency *β-θ* coupling is specifically suggested to support syntactic predictions^48,52^, while other sets of results have shown a modulation of the *δ*-band spectral components by combinatorial variables^53^. More research is needed to address the different models directly, and to further bridge ROI results and spectral analyses.

In contrast to the syntactic effects, LATL semantic composition appeared ~ 275 ms after adjective onset (Fig. 5). Here, we tackled two questions: (i) Does form typicality (the degree to which a word’s visual form gives away its category) facilitate semantic composition? and (ii) Do conceptual specificity effects during composition depend on a word’s position or its role? We found a three-way interaction between noun type, adjective form typicality, and adjective definiteness (Fig. 5c–d).

All high form-typicality adjectives elicited composition effects, regardless of definiteness or noun specificity (two leftmost panels in Fig. 5d). Low typicality adjectives eliminated composition effects (except in one case, discussed below).

To note, while low typicality adjectives had 4 letters in their bare (masculine, indefinite) form, high typicality adjectives varied in length (bare form mean: 4.7 letters, SD‍ = ‍0.89 letters). But it is unlikely that this small difference explains LATL effects; if word length had any effect on composition, we would expect the opposite: longer adjectives hindering composition.

In Arabic, noun-adjective pairs combine readily, whereas noun-noun pairs do not. Thus, the more likely interpretation of the effect is that high-typicality adjectives, with their adjective-biasing visual form (see Methods), carry sufficient cues to enable early composition, whereas low-typicality adjectives, with their noun-biasing visual form, do not provide enough evidence for this early semantic composition to occur. Our results thus support the hypothesis that semantic composition during reading depends on the availability of visual cues regarding the categories of the words being read. Recent research suggests that one possibility is that visual information reaches the LATL via a ventral stream, which includes occipital and inferior temporal regions^54^, which have been shown to subserve reading comprehension^55,56^. Importantly, the basis of our form typicality manipulation is morphological (see Methods), and activity in the visual word form area, which is part of the ventral stream, is affected by morphological properties during comprehension^50,51^.

In the conditions where we found conceptual specificity effects (definite, low-typicality adjectives), it was two-word trials with low-specificity (‘*period*’)—but not high-specificity (‘*year*’)—nouns that elicited significantly more positivity than single-word trials. This is consistent with previous research showing the same pattern on head nouns in English word pairs^35,36^; thus, it is the noun’s role (main vs. modifier), rather than its position, that seems to dictate conceptual specificity effects on LATL composition.

To complete the examination of the LATL effects, we turn to what underlies the difference between indefinite and definite low-typicality adjectives (two rightmost panels in Fig. 5d). One possibility is that definite articles could constitute another, albeit weaker, form-based cue: a definite article necessarily implies a word is either a noun or an adjective (and not a verb, or other). Thus, for definite, low form-typicality adjectives, there are only partial visual cues for the word’s category.

The emerging picture from this and other studies is one where the occurrence of early LATL composition effects depends on the strength of the available visual evidence: if there is strong evidence supporting composition (e.g., high form-typicality adjectives following a noun), it takes place, and if there is strong evidence against composition (e.g., low form-typicality adjectives that look like nouns), it does not. However, if the evidence is inconclusive (as with our definite low-typicality adjectives), whether early LATL composition occurs is sensitive to other features and factors, such as conceptual specificity in this case. Of course, further studies are required to directly test this hypothesis. Previous studies have shown that LATL activity is modulated by complex interactions between two sets of variables (e.g., conceptual and logical, like negation^57^).

Finally, as one reviewer remarked, it is important to note that while MEG’s array of sensors allows us to estimate the cortical sources of neural activity, these estimates can carry errors compared with real neuronal activities, mainly due to leakage of activity between real and reconstructed neural patterns^58^. Thus, it would be beneficial to use similar designs to test hypotheses and replicate results using high spatial resolution methodologies, such as fMRI.

In sum, a minimally-contrastive, basic composition design that dissociates syntactic variables from visual, lexical, and conceptual variables—without resorting to unnatural stimuli—revealed two syntactic effects in the LPTL. By elimination of other possibilities, it is likely that one effect corresponds to predictive structure building, while the other reflects bottom-up syntactic composition. A spectral analysis revealed a greater overall increase in activity for syntactically complex—compared to simple—conditions in the lower *β* frequency range. We also found semantic effects in the LATL, modulated by form typicality and conceptual specificity. These results provide support for models that implicate the LPTL in structure-driven processing, and the LATL in meaning-driven processing.

## Methods

### Participants

We recruited 34 right-handed native Arabic speakers at New York University (NYU; 18) and NYU Abu Dhabi (NYUAD). NYU and NYUAD Institutional Review Boards independently approved the experiment, which we performed in accordance with relevant guidelines and regulations. Participants had normal or corrected-to-normal vision. They provided written informed consent and received compensation. We excluded 7 participants’ data because of excessive noise and/or poor quality. We further excluded 6 participants’ data for scoring below 60% on one or more task types (Fig. 1d). We used data from 21 participants (12 at NYU; mean age = 26.8 years, SD = 5.7 years; 11 identified as female).

### Materials

We created 36 experimental sets and three practice sets. Each set contained 20 conditions: twelve two-word conditions (3 syntactic × 2 conceptual specificity × 2 form typicality levels), and eight single-word conditions (nouns: 2 specificity × 2 definiteness levels; adjectives: 2 form typicality × 2 definiteness levels). In total, there were 720 trials. Half the sets had feminine nouns/adjectives (i.e., had a single-letter feminine suffix), and half had masculine ones (no suffix).

We used the Word Tree in Arabic Wordnet (v2.0)^59^ to find hierarchically-related noun pairs of the same gender (e.g., *period* dominates *year*). We designated hierarchically-dominant nouns as low conceptual-specificity.

Our form-typical adjectives are derived from nouns by adding a single-letter suffix (yellow marking in Fig. 5a). The likelihood of words with this suffix being adjectives is 78.9% (calculated using Arabic Gigaword corpus (v5.0), parsed with MADAMIRA^60^). Thus, the words’ form provides a strong ‘adjectivality’ visual cue. Untypical adjectives were of the template C_1_aC_2_**i:**C_3_, where C_*j*_ is the *j*^th^ consonant in a triconsonantal Arabic root [C_1_, C_2_, C_3_]; in Arabic, different roots can be substituted into the same template to form different words. The corpus likelihood of words of this template being adjectives is 29.4%: the words’ form ( ‘**i:**’ between C_2_ and C_3_; orange marking in Fig. 5a) provides a strong visual cue against ‘adjectivality’, and in favor of ‘nouniness’.

### Task

Half the task items resulted in ‘good’ (grammatical and plausible) sentences, and half in ‘bad’ sentences, of which half had grammatical violations, and half had plausibility violations (Fig. 1d). We counterbalanced presence of task items and types of resulting sentences, across conditions.

### Stimuli presentation

We divided stimuli into 10 blocks (6 two-word, 4 single-word), using a Latin square design. Each word appeared only once per block (e.g., ‘*chair purple’* and ‘*the-throne the-purple*’ appeared in different blocks). For each participant, we randomized block order and trial order within blocks.

Each trial’s elements appeared on screen for 300 ms, followed by a 300 ms blank screen. Trials began with a fixation cross, followed by the first word. For two-word trials, the second word followed. On non-task trials, a symbol (<O>) appeared and participants pressed either button to continue (Fig. 1b). On task trials, the task sentence appeared until participants responded. To avoid entrainment to presentation frequency, after each trial a blank screen appeared with a random uniformly-distributed duration (466.66–700 ms).

A projector relayed the image onto a screen inside the Magnetically Shielded Room (MSR); visual angles across both systems were 0.7° vertically. For presentation, we used PsychoPy2^61^ (v1.84.2) and the Arabic Text Reshaper package (https://github.com/mpcabd/python-arabic-reshaper). Content appeared in white against gray. Explanation screens appeared first, followed by practice sets and experimental stimuli. The experiment lasted ~ 55 min.

### Data acquisition

Before the experiment, we digitized participants’ head-shape using a hand-held FastSCAN laser scanner (Polhemus, VT, USA) for later co-registration (i.e., aligning brain, sensors, and head-shape within the MSR; see ‘MEG data pre-processing’ below). We marked and digitized five points on the head: center, left, and right of forehead, and one anterior of each auditory canal. Inside the MSR, we placed marker coils on digitized points to localize the participant’s head relative to the MEG sensors. Marker measurements obtained right before and after the experiment measured overall movement. During the experiment, we acquired MEG data using 157- (NYU) and 208-channel (NYUAD) axial gradiometer systems (Kanazawa Institute of Technology, Kanazawa, Japan), with a 1000 Hz sampling rate, and an online 200 Hz low-pass filter. We collected structural Magnetic Resonance volumes from 5 participants using NYUAD’s MRI facility (3T MAGNETOM Prisma, Siemens).

### MEG data pre-processing

We noise-reduced data using the Continuously Adjusted Least Squares Method (CALM^62^; MEG160 software v2.004A—Yokogawa Electric Corporation and Eagle Technology Corporation, Tokyo, Japan), which discounts noise recorded in reference channels, located inside the MSR away from the brain. We imported data into MNE-Python^63^ (v0.16), and band-pass-filtered them between 1 and 40 Hz. We overrode bad channels (flat/excessively noisy; NY: min = 7, max = 11, median = 8; AD: min = 7, max = 16, median = 9) and interpolated missing data using remaining sensors. Then, we applied an independent component analysis (ICA) algorithm, to identify and remove system-characteristic or identifiable noise components (e.g., eyeblinks, heartbeats) based on visual inspection. Afterwards, we segmented data into epochs, -100–1200 ms relative to first word onset (for semantic composition analysis, single-adjective epochs were epoched relative to fixation cross onset, to temporally align adjectives across one- and two-word blocks; Fig. 5a). Finally, we baseline-corrected epochs using the first 100 ms, and rejected epochs containing amplitudes exceeding 3000 fT (NYU) or 2000 fT (NYUAD; difference due to ambient magnetic noise differences between systems/cities.) On average, we lost 0.27% (SD = 0.36%) of trials at this stage.

For co-registration, we scaled the FreeSurfer average brain^64^ to match participants’ head-shape, and created a source-space mesh of 2,562 vertices per hemisphere. For participants with structural MR volumes, we used those for co-registration instead of FreeSurfer’s average. Using the Boundary Element Model method, we calculated a forward solution from vertices, then estimated the inverse solution per trial, per subject, assuming an SNR value of 1. We constrained dipoles along the direction orthogonal to local cortical patches, yielding a signed activation estimate. The inverse solution resulted in a noise-normalized Dynamic Statistical Parameter Map (dSPM^65^).

### ROIs

We averaged dSPM values within each ROI’s sources. For syntactic analysis ROIs, we used MNI-coordinate seeds from a study reporting clusters in all four regions^9^ (Table 1). We grew seeds up to a 40 mm radius (LIFC: 30 mm) without overlap (Fig. 2a). For right-hemispheric homologues, we reversed seeds’ *x*-coordinate sign, growing the labels similarly.

**Table 1:** MNI coordinates of ROI seeds.

ROI	Seed MNI coordinates
LPTL	(− 51, − 39, 3)
LATL	(− 48, 15, − 27), (− 54, − 12, − 12)
LIFC	(− 45, 33, − 6), (− 51, 21, 21)
LAG	(− 39, − 57, 18)

For semantic analysis ROIs, we picked an MNI seed on the cortical surface of the left temporal pole ((– 57, – 2, – 33); RATL: reversed *x-*coordinate), and grew it up to 40 mm, roughly covering the anterior half of the lobe (Fig. 5b).

### Statistical models and analyses

We built and estimated regression models using R (v3.6.1) and RStudio (v1.2.5001). Linear mixed effects models depended on the question, but included these fixed effects where possible/relevant: trial onset-time from experiment start (log-transformed, z-scored), gender (factor: masculine, feminine), Zipf frequency of nouns/adjectives^66^ (i.e., log_10_(frequency/billion words); z-scored). Additionally, we included the variables of interest (see Results). Random effects included intercepts per participant; more complex random effects structures resulted in non-convergence.

Analyses regressed dSPM values at each timepoint using two nested models; reduced models always left out our variable(s) of interest. Model comparison produced timecourses of *χ*^2^-distributed statistics quantifying the model’s improvement due to variable(s) of interest. We identified clusters of contiguous timepoints with statistics exceeding the 95th-percentile threshold of χ^2^ distribution. We defined cluster size as the sum of its χ^2^ values. Per ROI, we chose the biggest cluster’s size as the significance-determining statistic.

We determined significance using a cluster-based (Monte Carlo) permutation test^67^. We randomly shuffled condition labels per participant 10,000 times, re-comparing models to retrieve the biggest cluster per ROI. For each ROI, we compared the real cluster against a distribution of 10,000 simulated clusters. ROI *p*-values equal the proportion of permutations with clusters larger than the real cluster, within test window. We corrected *p*-values for multiple comparisons across ROIs using the False Discovery Rate (FDR) procedure^68^. Effects are significant if corrected *p*-values < 0.05.

For pairwise contrasts, we regressed averaged ROI activity within cluster against the full model, and compared model-based estimated marginal means, correcting for multiple comparisons using multiplicity adjustment.

### Spectral analysis

Using MNE-Python, we conducted spectral analyses on the MEG sensor data in two-word trials. Using Morlet wavelets (4 cycles globally), we calculated time–frequency representations of each epoch, for each participant and sensor independently. As we had a specific hypothesis about the *β-*range, we limited the frequency domain to 8–32 Hz, with 1 Hz steps. After spectral decomposition, we downsampled time–frequency representations by 5 along the time domain, for computation efficiency. We calculated the log-ratio of activity increase per epoch, compared to baseline (-100–0 ms). For each condition and subject, we averaged activity profiles across all sensors and trials, resulting in a three-dimensional matrix (subject, time, frequency) per condition.

We performed spectro-temporal clustering analyses, comparing the three condition pairs: a time–frequency point contributed to a cluster if the local, one-tailed paired *t*-test statistic corresponded to a value more extreme than the 95% percentile. We then ran 10,000 permutation analyses, yielding 10,000 clusters. If the biggest real cluster was larger than 95% of permutation clusters, we concluded there is a significant difference between conditions in test window. We corrected for three comparisons using FDR.

Finally, we repeated this analysis for time–frequency representations calculated based on average evoked (not single-trial) responses per subject and condition. This eliminates oscillatory contributions to the time–frequency representations, and allows us to qualitatively ensure that effects in single-trial data reflect genuine oscillatory signatures, not phase-locked spectral components^27^.

## Supplementary Information

Supplementary Information

## Data Availability

The datasets generated and analyzed during the current study are available from the corresponding author upon reasonable request.
