# Supplementary material for: Left posterior temporal cortex is sensitive to syntax within conceptually matched Arabic expressions
Source: Sci Rep. 2021 Mar 30;11:7181. doi: 10.1038/s41598-021-86474-x (PMC8010046; doi:10.1038/s41598-021-86474-x)
Supplement: Supplementary file 1 — Supplementary Information [file 41598_2021_86474_MOESM1_ESM.pdf]

Left posterior temporal cortex is sensitive to syntax within conceptually-matched Arabic expressions

Suhail Matar, Julien Dirani, Alec Marantz, & Liina Pylkkänen

Supplementary Information

Supplementary Results

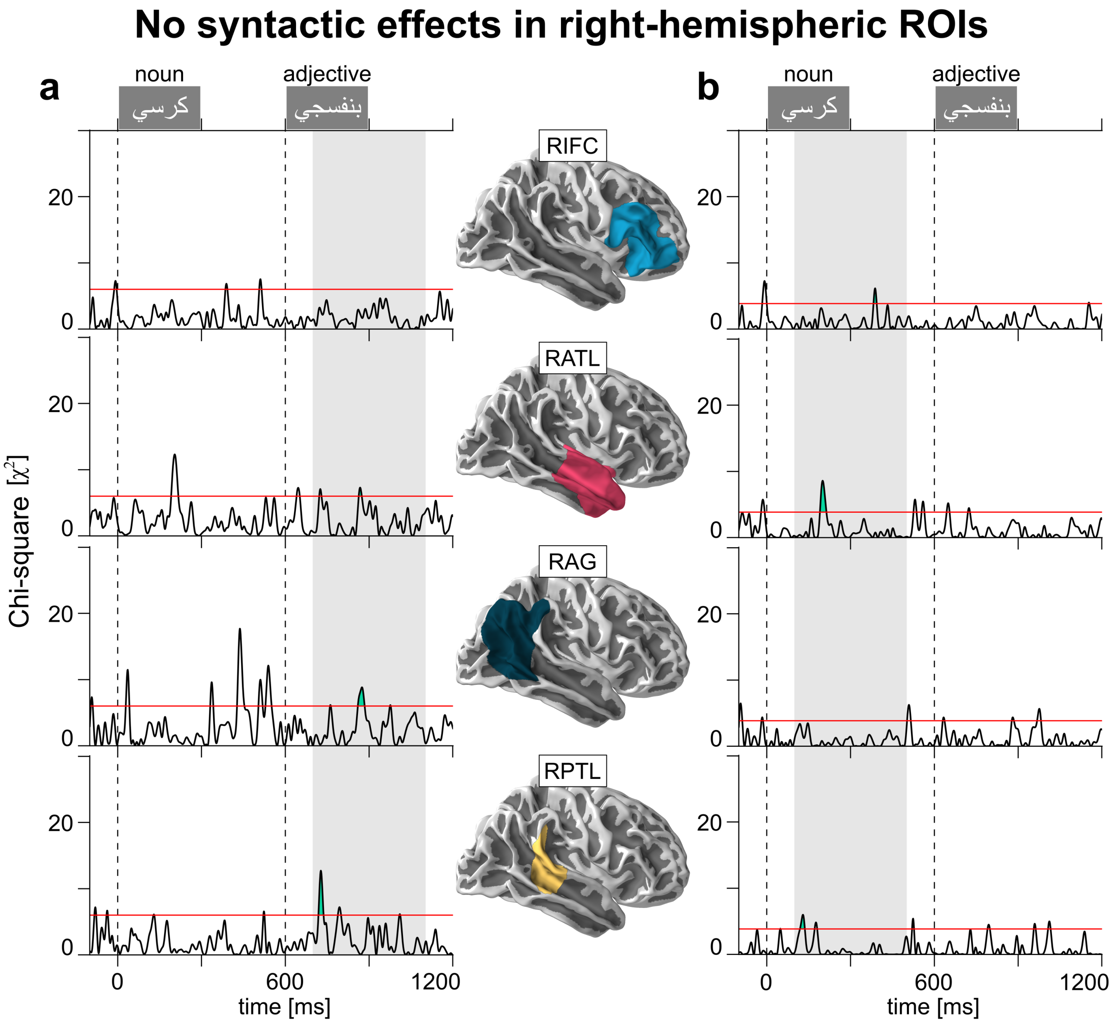

Supplementary Figure S1. Results of syntactic analysis in right-hemispheric ROIs. (a) Plots show results of likelihood ratio tests, comparing models with and without the syntactic factor, for each cortical ROI. Gray bar indicates test time window (700–1100 ms). Horizontal red lines indicate cluster-forming thresholds (95th-percentile of $\chi^2(2)$). Filled green areas indicate suprathreshold clusters. (b) Model comparison results assessing effect of noun definiteness in noun window (100–500 ms). Red lines indicate 95th-percentile of $\chi^2(1)$.

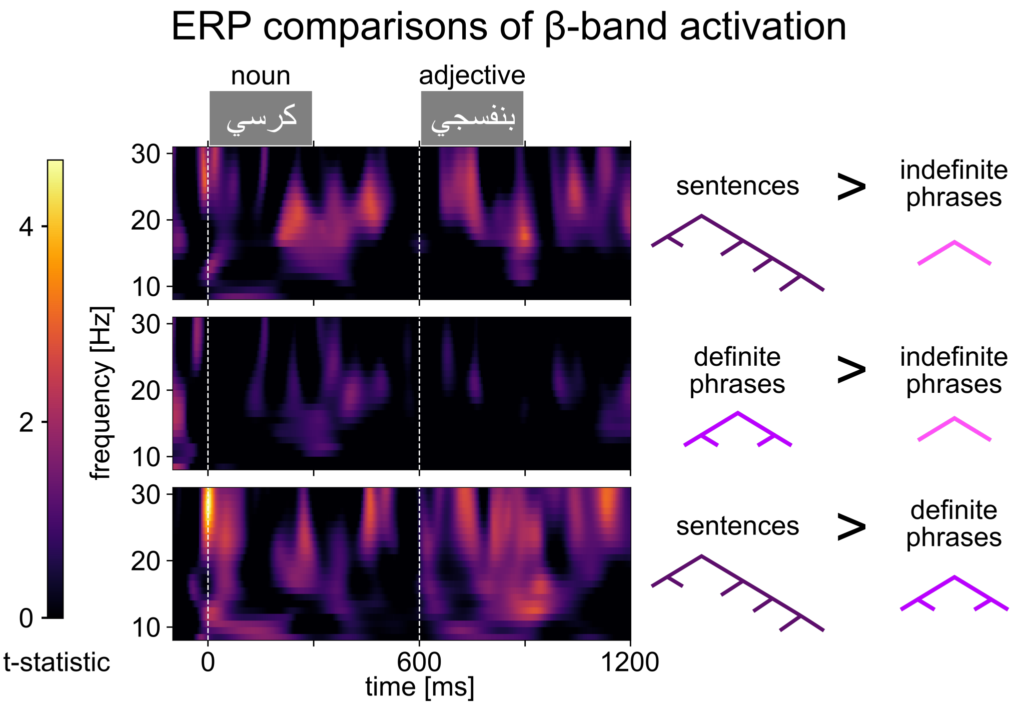

Supplementary Figure S2. Pairwise comparisons of time-frequency representations of increased activity in two-word trials compared to baseline, calculated based on the average evoked response per condition, per participant. Color bar shows *t* statistic values. White dashed lines represent word onsets.

Supplementary Table S2. Pairwise comparisons of LATL effects. We performed three pairwise comparisons per each level of adjective definiteness and form typicality. *p*-values are corrected for multiple comparisons. Bold *p*-values indicate statistical significance (<0.05).

Adjective form typicality	Adjective definiteness	Noun type contrasts	Mean estimate [dSPM], *t*-statistic (*p*-value)
Typical	Definite	Low-spec – None	$9.13\cdot10^{-3}$, 2.897 **(.0105)**
		High-spec – None	$8.28\cdot10^{-3}$, 2.629 **(.0233)**
		Low-spec – High-spec	$0.85\cdot10^{-3}$, 0.271 (.9604)
Typical	Indefinite	Low-spec – None	$11.5\cdot10^{-3}$, 4.213 **(.0001)**
		High-spec – None	$9.86\cdot10^{-3}$, 3.615 **(.0009)**
		Low-spec – High-spec	$1.64\cdot10^{-3}$, 0.738 (.7408)
Untypical	Definite	Low-spec – None	$9.47\cdot10^{-3}$, 3.011 **(.0074)**
		High-spec – None	$6.19\cdot10^{-3}$, 1.966 (.1207)
		Low-spec – High-spec	$3.29\cdot10^{-3}$, 1.045 (.5481)
Untypical	Indefinite	Low-spec – None	$3.97\cdot10^{-3}$, 1.456 (.3124)
		High-spec – None	$2.05\cdot10^{-3}$, 0.751 (.7328)
		Low-spec – High-spec	$1.92\cdot10^{-3}$, 0.863 (.6353)

Syntactic entropy calculations. Using Arabic Gigaword corpus (v5.0) –parsed and analyzed with MADAMIRA[1]– we calculated corpus-based syntactic entropy using the formulas:

$$(1)\ H_{init}(W_1) = -\sum_{w \in W_1} P(w|init) \log_2 P(w|init)$$

$$(2)\ H_N(W_2) = -\sum_{w \in W_2} P(w|init, N) \log_2 P(w|init, N)$$

$$(3)\ H_A(W_3) = -\sum_{w \in W_3} P(w|init, N, A) \log_2 P(w|init, N, A)$$

Before a trial begins, entropy is a function of the probability distribution of the syntactic category of any sentence initial word (W_1; equation (1)). On the noun, entropy is a function of the probability distribution of the syntactic category of the second word (W_2), given an initial noun (equation (2)). On the adjective (the second word), it is a function of the probability distribution of the third word (W_3), given an initial noun-adjective pair (equation (3)). Given our design, we treated indefinite and definite nouns as separate categories (same for adjectives). Supplementary Table S3 provides syntactic entropy values for definite and indefinite nouns and adjectives within our design.

Supplementary Table S3. Corpus-based entropy values for each word in our design.

<table>
<tr><th>Initial word</th><th>$H_{init}(W_1)$</th><th>Noun type</th><th>$H_N(W_2)$</th><th>Condition (Noun + Adj type)</th><th>$H_A(W_3)$</th></tr>
<tr><td rowspan="3">Sentence inception</td><td rowspan="3">1.925</td><td>Initial indefinite noun</td><td>2.421</td><td>Indefinite phrase
(indefinite noun + indefinite adjective)</td><td>2.336</td></tr>
<tr><td rowspan="2">Initial definite noun</td><td rowspan="2">2.141</td><td>Definite phrase
(definite noun + definite adjective)</td><td>2.234</td></tr>
<tr><td>Sentence
(definite noun + indefinite adjective)</td><td>2.308</td></tr>
</table>

Syntactic entropy reduction calculations. We used entropy values (Supplementary Table S3) to calculate entropy reduction, defined as follows:

$$(4)\ \Delta H(W_n) = \begin{cases} H(W_n) - H(W_{n-1}), & H(W_n) < H(W_{n-1}) \\ 0, & H(W_n) \geq H(W_{n-1}) \end{cases}$$

For each word, entropy reduction is zero if entropy increases, compared to the previous word; otherwise, entropy reduction is defined as the difference in entropy between the current word and the previous word. Supplementary Table S4 provides syntactic entropy reduction values for each part of our design.

Supplementary Table S4. Corpus-based entropy reduction values for each word in our design.

Noun type	*ΔH(W1)*	Condition (Noun + Adj type)	*ΔH(W2)*
Initial indefinite noun	0	Indefinite phrase (indefinite noun + indefinite adjective)	0.085
Initial definite noun	0	Definite phrase (definite noun + definite adjective)	0
		Sentence (definite noun + indefinite adjective)	0

Syntactic surprisal calculations. Using the Arabic Gigaword corpus, we calculated corpus-based syntactic surprisal using the formulas:

$$(5)\ S_N = -\log_2(P(N|init))$$

$$(6)\ S_A = -\log_2(P(A|init, N))$$

For nouns, surprisal is a function of the probability of the initial word in a sentence being a noun. For adjectives, surprisal is a function of the probability of an adjective following an initial noun. Supplementary Table S5 specifies syntactic surprisal values for definite and indefinite nouns and adjectives within our design.

Supplementary Table S5. Corpus-based surprisal values for each word in our design.

Noun type	S_N	Condition (Noun + Adj type)	S_A
Initial indefinite noun	3.183	Indefinite phrase (indefinite noun + indefinite adjective)	3.226
Initial definite noun	4.518	Definite phrase (definite noun + definite adjective)	1.362
		Sentence (definite noun + indefinite adjective)	5.886

References

1. Pasha, A. *et al.* MADAMIRA: A Fast, Comprehensive Tool for Morphological Analysis and Disambiguation of Arabic. in *Lrec* 1094–1101 (2014).
